# Predictors of postpartum coparenting relationship among primiparous couples in Karachi, Pakistan

**DOI:** 10.3389/fpsyt.2026.1886830

**Published:** 2026-09-25

**Authors:** Salima Sulaiman, Aliyah Dosani, M. Sarah Rose, Rozina Somani, Gulnar Ali

**Affiliations:** 1 School of Nursing, Brock University, St. Catharines, ON, Canada; 2 School of Nursing and Midwifery, Mount Royal University, Calgary, AB, Canada; 3 Department of Community Health Sciences, Cumming School of Medicine, University of Calgary, Calgary, AB, Canada; 4 O’Brien Institute of Public Health, University of Calgary, Calgary, AB, Canada; 5 University of Calgary, Calgary, AB, Canada; 6 College of Nursing, University of Saskatchewan, Saskatoon, SK, Canada; 7 Department for Social Work, Counselling and Social Care, School of Childhood and Social Care, University of East London, London, United Kingdom

**Keywords:** anxiety, coparenting relationship, depression, mothers, partner support, partners, relationship satisfaction, depressive symptoms

## Abstract

**Introduction:**

The postpartum coparenting relationship is an important determinant of parental well-being, relationship functioning, and child development during the transition to parenthood. However, most studies combine mothers’ and partner’s data, limiting the identification of parent-specific predictors of the postpartum coparenting relationship. This study examined predictors of the postpartum coparenting relationship among primiparous Pakistani couples in Karachi by analyzing mothers’ and partners’ data separately.

**Methods:**

The full study recruited primiparous mothers and their partners through convenience sampling during the first or second trimester from a tertiary care hospital in Karachi, Pakistan. The analysis utilized data from 101 couples who completed questionnaires at 12-weeks postpartum. Couples were assessed for anxiety symptoms, depressive symptoms, relationship satisfaction, partner support, and coparenting relationship using the State-Trait Anxiety Inventory, Edinburgh Postnatal Depression Scale, Patient Health Questionnaire-9, Revised Dyadic Adjustment Scale, Postpartum Partner Support Scale, and Coparenting Relationship Scale, respectively, during pregnancy and at 12-weeks postpartum. Anxiety and depressive symptoms were also assessed at 4–6-weeks postpartum. Multivariable regression models were used to examine predictors of postpartum coparenting relationship separately for mothers and partners.

**Results:**

The mean age of mothers was 26.9 years and 30.6 years for partners. Individual regression model suggested that mothers’ ethnicity (p < 0.001), partners ethnicity (p < 0.001), mothers’ education (p < 0.001), and partner’s education (p < 0.001) were individually and significantly associated with mothers’ postpartum coparenting relationship at 12-weeks postpartum. In the multivariable regression model, mothers’ postpartum coparenting relationship was significantly predicted by her postpartum relationship satisfaction (p = 0.023), postpartum partner support (p < 0.001), and partners’ antenatal depressive symptoms (p = 0.016). Similarly, the multivariable regression model showed that partner’s postpartum coparenting relationship was predicted by mothers’ postpartum coparenting relationship (p = 0.009), partners’ antenatal coparenting relationship (p = 0.001), partners’ postpartum relationship satisfaction (p = 0.021), and partners’ postpartum anxiety at 12-weeks (p = 0.005).

**Discussion:**

Findings support the view that coparenting is embedded within broader relational and sociocultural systems, where individual well-being, relationship quality, and reciprocal influences shape early postpartum family functioning. Timely screening for anxiety symptoms, depressive symptoms, and coparenting relationship in clinical settings for both parents may facilitate early identification and interventions to strengthen partner support and promote family well-being.

## 1 Introduction

The transition to parenthood is widely recognized as a major developmental life event that brings profound psychological, relational, and behavioral changes for couples (1). The arrival of a child requires individuals to adapt to new identities and responsibilities as mothers and partners, often reshaping their sense of self, self-esteem, and overall role functioning (1, 2). During this period, adults integrate caregiving demands with pre-existing personal, occupational, and relational roles, which can create both opportunities for growth and sources of strain. Although parenthood is often associated with joy and fulfillment, the adjustment process can also be accompanied by considerable stress and vulnerability. Many parents report heightened emotional distress during the perinatal period, including increased stress, anxiety, and depressive symptoms (3–7). These challenges may be intensified by sleep disruption, physical recovery following childbirth, financial pressures, changing routines, and uncertainty regarding infant care (8, 9).

Beyond individual adaptation, the transition to parenthood also has important implications for the couple’s relationship. Partners frequently experience changes in relationship satisfaction, intimacy, communication patterns, and emotional connection during this time (10–12). Common difficulties include negative or insufficient communication, conflict regarding household or parenting responsibilities, and reduced sexual intimacy, all of which may negatively affect partner attachment and relationship quality (11, 13). As couples respond to these evolving demands, they often undergo a broader structural reorganization in which roles, expectations, and responsibilities are renegotiated to accommodate the needs of the infant. Overall, the transition to parenthood represents a complex period of adjustment that can shape parental well-being, couple functioning, and long-term family relationships.

Central to this reorganization is the development of the coparenting relationship, referring to the ways in which parents work together in their shared roles to raise their child (14). The coparenting relationship has been associated with a range of positive outcomes for both children and parents (15, 16). For children, supportive coparenting has been linked to better mental health, higher self-esteem, stronger academic performance, and improved overall adjustment, including greater soothability, attention span, and sleep quality (15, 16). For parents, a positive coparenting relationship has been associated with greater coparenting support, higher marital satisfaction, improved mental well-being, and stronger parenting efficacy (15). Despite growing research on coparenting, uncertainty remains regarding whether coparenting begins during pregnancy through expectations and planning (14, 17–19), or whether it fully emerges after birth through shared caregiving experiences (20). Understanding how couples navigate the transition to parenthood is therefore essential for identifying factors that promote a healthy coparenting relationship, effective family functioning, and long-term parental and child well-being.

Evidence supporting a prenatal origin of the coparenting relationship suggests that expectant couples begin developing shared representations of their unborn infant and anticipating changes in their lives as they prepare for parenthood (14, 18, 19). During pregnancy, prospective parents often imagine how they will divide parenting responsibilities, support one another, and adapt their relationship and daily routines following the birth of their child (19). For example, Sevon (18) reported that expectant women talked optimistically and positively about their partner, relationship, commitment, and future parental involvement during pregnancy. Supporting this perspective, Delmore-Ko and colleagues (21) found that women who felt prepared to welcome their child reported lower stress and higher self-esteem, which were associated with greater competence in child-rearing. These anticipatory processes may represent the early foundations of coparenting relationship, as couples begin negotiating expectations regarding caregiving roles, decision-making, and family functioning before the infant arrives.

On the contrary, not all scholars agree that coparenting can be meaningfully evaluated before birth. Van Egeren and colleagues (20), for example, argued that the coparenting relationship cannot be fully assessed until after childbirth, as prenatal expectations and intentions may not accurately reflect actual parenting behaviors once couples encounter the realities of infant care. The demands of caregiving, sleep deprivation, infant temperament, and postpartum recovery (8, 9) may substantially alter previously held expectations and relational dynamics. These differing perspectives highlight an important gap in the literature regarding the extent to which prenatal coparenting expectations translate into postnatal coparenting experiences, underscoring the need for longitudinal research to inform early, preventive interventions.

Empirical studies examining prenatal coparenting behaviors provide further insight into this debate. Although limited in number, available studies suggest that prenatal interaction patterns may predict the quality of postnatal coparenting (17, 22, 23). For example, expectant partners’ intuitive parenting behaviors, together with those of women, predicted partners’ later involvement in developmentally appropriate interactions with their infants at three months postpartum (23). In contrast, couples who displayed antagonistic behaviors during pregnancy, such as competition and verbal sparring, often continued these patterns after childbirth (17). Additional evidence indicates that prenatal coparenting behaviors, including playfulness and partners’ intuitive parenting behaviors, may strongly predict later postnatal coparenting dynamics (22). These findings suggest that relational patterns established during pregnancy may offer an early indicator of how couples will function together as parents after birth.

However, most of this research has been conducted in Western contexts and has relied primarily on observational methods, such as the Prenatal and Postnatal Lausanne Trilogue Play (PLTP), to assess triadic interactions among both parents and the infant. This structured observational procedure is designed to observe and code family triadic interactions before and after birth (17, 22, 23). Such approaches are valuable because they include both parents and assess the coparenting relationship as a shared family process rather than solely individual perspectives. However, despite these contributions, the PLTP has a few notable methodological limitations. First, during the prenatal phase, the absence of a real infant may limit the validity of the observed interactions (22). Specifically, parental behaviors are elicited in a simulated context rather than in response to actual caregiving demands. Secondly, interpretation of the observational data may be influenced by researcher subjectivity, which can introduce variability in how behaviors are coded and understood. Furthermore, parents’ behaviors may be influenced by the awareness of being observed, potentially leading to modified or socially desirable interactions rather than fully naturalistic coparenting dynamics. Third, the PLTP has limitations in capturing individual expectations regarding coparenting, as the method primarily focuses on observed triadic interactions rather than each parent’s personal beliefs, concerns, or anticipated roles within the coparenting relationship (22). Consequently, the individual voices and subjective perspectives of mothers and partners have been largely underrepresented in PLTP-based coparenting research.

Building on the limitations of observational approaches such as the PLTP, emerging research has begun to incorporate self-reported measures of coparenting expectations. To the authors’ knowledge, only one recent study conducted in Finland (24) has examined the association between prenatal and postnatal coparenting separately for mothers and partners using individual reports. Findings from this study indicated that parents’ prenatal expectations of coparenting predicted their own level of postnatal coparenting, suggesting that individual cognitive and emotional representations formed during pregnancy play an important role in shaping their later coparenting experiences. This study highlights the value of moving beyond solely observational methods to include parents’ subjective perceptions when examining coparenting relationship.

Despite growing evidence that prenatal coparenting representations and behaviors may shape postnatal coparenting quality, the literature remains limited and is primarily concentrated in Western contexts. As a result, little is known about how these processes unfold in low- and middle-income countries (LMICs), where cultural norms, gender roles, family structures, and social support systems may differ substantially. Expanding research into LMIC settings is therefore essential to determine the cultural relevance, applicability, and broader generalizability of current theoretical and empirical models of perinatal coparenting.

The context of LMICs present unique considerations, where parenting is perceived and practiced differently due to gender-based norms surrounding the distribution of housework and infant care (25). In many such settings, caregiving is primarily seen as the mother’s responsibility, while partners are more often positioned as financial providers or decision-makers rather than active daily caregivers (25, 26). Women face gender-specific role restrictions, and are primarily responsible for housework and infant care, often carrying excessive unpaid workloads with limited autonomy (25). Partners’ participation in household and caregiving activities is limited, typically involving minimal engagement in daily chores, and focused primarily on interacting with the infant, or teaching and transmitting family values (27). These gendered norms reinforce unequal caregiving roles within families and shape how coparenting is experienced and practiced in LMIC contexts.

Given these gender differences, the coparenting relationship may be perceived differently in the context of LMIC, particularly in Pakistan. This study aimed to examine predictors of the postpartum coparenting relationship separately for mothers and partners among primiparous Pakistani couples in Karachi, Pakistan. To our knowledge, this is the first study to explore the coparenting relationship in a LMIC setting using validated and reliable tools to measure the coparenting relationship during both the prenatal and postnatal period.

## 2 Materials and methods

### 2.1 Participants

The full study recruited primiparous mothers and their partners during the first or second trimester from the obstetrics and gynecology clinic at the Aga Khan University Hospital in Karachi, Pakistan between September 2018 and February 2020. Participants who provided baseline information and completed the 12-weeks postpartum assessment were eligible for inclusion in the present study. The sample consists of 101 couples (101 mothers and 101 partners) who completed the questionnaires at all time points, and their data were included in the analysis for this paper. Reasons for loss to follow-up at both the 4–6-weeks and 12-weeks assessments included non-response to telephone calls, failure to attend the scheduled postpartum clinic visit (for the 4–6-weeks assessment), and stillbirth. Please refer to the **Supplementary Figure 1** STROBE diagram for participants’ flow. Detailed inclusion and exclusion criteria have been reported elsewhere (28).

### 2.2 Study ethics

Prior to data collection, ethical approval was obtained from the Ethics Research Committee at Aga Khan University Hospital (5329-obs-ERC-18), the Health Sciences Research Ethics Board at University of Toronto (ID: 00015630-V0001.000R), Research Ethics Board at Brock University (22-102) and the Human Research Ethics Board at Mount Royal University (ID: 103237). Written informed consent was obtained from all participants.

### 2.3 Procedure

A research assistant (RA) approached couples in the waiting area of the clinic during their scheduled antenatal visits to explain the study. Those couples that met the eligibility criteria were invited to participate. Informed consent was obtained prior to completing several questionnaires. At recruitment, all couples completed the Edinburgh Postnatal Depression Scale (EPDS), Patient Health Questionnaire-9 (PHQ-9), State-Trait Anxiety Inventory (STAI), Coparenting Relationship Scale (CRS), Revised Dyadic Adjustment Scale (RDAS), and Postpartum Partner Support Scale (PPSS) to assess antenatal characteristics. The same measures including EPDS, STAI, PPSS, CRS, and RDAS were administered again to both parents at 12 weeks postpartum by telephone to assess postnatal outcomes. In addition, both parents completed the EPDS and STAI at 4–6 weeks postpartum during the routine follow-up visit in the clinic to capture early postpartum depressive and anxiety symptoms. If the visit was missed, the RA attempted to complete the assessment by telephone.

### 2.4 Measures

1. Depressive Symptoms Assessment: the depressive symptoms were assessed using the EPDS and PHQ-9 scales. The EPDS (29) is a 10-item self-report measure designed to assess depressive symptoms in the past weeks. Each item is rated on a four-point Likert scale, with total scores ranging from 0 to 30. Higher scores indicate more severe depressive symptoms. The EPDS has demonstrated a Cronbach’s alpha ranging from 0.73 to 0.87 (29). The EPDS were previously validated in Pakistani pregnant women and male partners, with overall Cronbach Alpha of 0.83 and 0.80, respectively (28, 30), indicating acceptable reliability. Women completed the EPDS as an eligibility criterion at the time of recruitment and their scores were <12, shows no depressive symptoms. The PHQ-9 (31) is a nine item self-report questionnaire used to assess depressive symptoms over the past two weeks. Each item is scored on a four-point Likert scale ranging from 0 (“Not at all”) to 3 (“Nearly every day”), with total scores ranging from 0 to 27. Higher scores indicate greater depressive symptoms. Using a cut-off score of 10, the PHQ-9 has a sensitivity of 88% and a specificity of 88% for major depressive symptoms. The PHQ-9 was previously validated in Pakistani male partners of pregnant women with overall Cronbach Alpha of 0.79 (28) and is widely used in clinical and research settings to screen for depressive symptoms (31).
2. Anxiety Symptoms Assessment: the anxiety symptoms were measured using the STAI (32), a 20-item self-report instrument that measures anxiety. Each item scored on a four-point Likert scale with a total score ranging from 20 to 80. Couples who score > 40 are likely to be suffering symptoms of anxiety. The internal consistency of the STAI ranges from 0.91 to 0.95.
3. Coparenting Relationship Assessment: the coparenting relationship was assessed using the CRS. The CRS (33) is a thirty-five item self-report questionnaire that measures coparental quality. Each item is scored on a six or seven-point Likert scale, with total scores ranging from 0 to 210. Higher scores indicate a positive coparenting relationship. It has good reliability and strong stability with Cronbach Alpha ranging from 0.91 to 0.94 across genders (33). For use during the antenatal period, the wording of the tool was adapted to reflect expected rather than actual coparenting experiences. For example, the item “My partner and I have the same goals for our child” was modified to “My partner and I will have the same goals for our child” to capture prenatal coparenting expectations.
4. Relationship Satisfaction Assessment: relationship satisfaction was measured using the RDAS. The RDAS (34) is a 14-item self-report, measures couples’ relationship satisfaction. Each item is rated on a six-point Likert scale with a total score ranging from 0-69. The cut-off score for the RDAS is 48, such that scores of 48 and above indicate relationship satisfaction and scores of 47 and below indicate relationship dissatisfaction. The Cronbach Alpha for the RDAS is 0.90 (34).
5. Postpartum Partner Support Assessment: the postpartum partner support was measured using the PPSS. The PPSS (35) is a 20-item self-report questionnaire used to assess partner support over the past four weeks. Responses are recorded on a 4-point Likert-type scale, with higher scores reflecting greater perceived partner support. The Cronbach Alpha is 0.96 (35). For use during the antenatal period, the wording of the tool was adapted to reflect expectation of the partner support after the birth of a baby. For example, “Respects the decisions I make as a parent” was modified to “Will respect the decisions I make as a parent”. All tools were translated into Urdu and piloted with couples to ensure cultural appropriateness.

### 2.5 Statistical analysis

This was an exploratory analysis, since there were no priori hypotheses and the sample size, determined by the original study, was relatively small. The original sample used 95% one side CI (lower bound) and precision of 0.10 to estimate 65% feasibility of the priori criteria established for the larger study, 88 couples (mothers and partners) were required (36). An attrition rate of approximately 20% was considered (37); therefore, the adjusted sample size was increased to 106 couples per group, with a total of 212 couples (212 mothers and 212 partners). Six couples experienced miscarriage and subsequently withdrew from the study, resulting in a total sample of 206 couples (206 mothers and 206 partners). Descriptive statistics were presented as means, standard deviations, and quartiles for continuous variables, and as frequencies and percentage for categorical variables. The correlation between all the variables was calculated. Assessing correlations was important for two reasons. First, correlation analysis helps determine whether variables measure the same or related constructs. Second, examining correlations is essential when developing regression models, as highly correlated variables (i.e., multicollinearity) should not be included simultaneously in the same model (38). Including highly correlated predictors may result in neither variable appearing statistically significant when entered together, even though each may demonstrate significance when analyzed independently (38).

The variables identified in the univariable analyses (p < 0.20) were included in an initial multivariable model. The model was then iteratively refined through manual evaluation of predictor importance, clinical relevance, confounding, multicollinearity, model fit, and regression diagnostics to obtain the final parsimonious model. Automated stepwise variable selection procedures were not used. Model assumptions were assessed using residual diagnostic plots (residuals versus fitted values, normal Q-Q plots, and scale-location plots), together with formal tests of residual normality (Shapiro-Wilk), homoscedasticity (Breusch-Pagan), and autocorrelation (Durbin-Watson). Multicollinearity was assessed using variance inflation factors (VIF) and tolerance statistics. Influential observations were evaluated using leverage and Cook’s distance. Because formal assumption tests may detect minor departures that have little practical impact and increase the chance of spurious results due to problems of multiple hypothesis testing, graphical diagnostics and sensitivity analyses using heteroscedasticity-robust standard errors were used to evaluate the robustness of the regression results.

## 3 Results

### 3.1 Participants

The demographic characteristics of the sample are presented in **Table 1**. The mean age of mothers was 26.9 years, whereas the mean age of partners was 30.6 years. At recruitment, women were, on average, 16.6 weeks pregnant. The majority of mothers held a bachelor’s degree, while most partners had completed a master’s degree or PhD.

**Table 1:** Demographic characteristics of women and partners.

Variables	Mothers data N=101 n (%)	Partners data N=101 n (%)
Age [Mean (SD)]	26.9 (3.2)	30. 6 (3.3)
Duration of Marriage in Months [Mean (SD)]	18.5 (17.1)	
Weeks Pregnant [Mean (SD)]	16.6 (5.2)	
Education		
High School (Grade 11-12)	7 (7)	4 (4)
Bachelor’s	61 (60)	47 (47)
Graduate (Master’s and PhD)	33 (33)	50 (50)
Marriage type		
Arranged	65 (65)	
Love	35 (35)	
Planned Pregnancy	87 (86)	
Family type		
Extended	47 (47)	
Nuclear	54 (53)	
Ethnicity		
Memon	6 (6)	4 (4)
Sindhi	11 (11)	16 (16)
Katchi	5 (5)	4 (4)
Gujrati	9 (9)	12 (12)
Punjabi	15 (15)	17 (17)
Pathan	3 (3)	1 (1)
Others	18 (18)	13 (13)
Urdu	34 (34)	34 (34)
Birth outcome*		
Term baby	82 (82)	
Preterm Baby	14 (14)	

*5 data missing.

### 3.2 Mothers

The correlations between all variables are reported (see **Supplementary Table 1**). Individual regression models were examined for demographic, antenatal, and postpartum variables predicting mothers’ postpartum coparenting relationship at 12-weeks postpartum (**see Supplementary Tables 2**-**4**). Demographic variables that individually, significantly predicted mothers’ postpartum coparenting relationship at 12-weeks were mothers’ ethnicity, partners ethnicity, mothers’ education and partner’s education (See **Supplementary Table 2**). Antenatal variables that individually, significantly predicted mothers’ postpartum coparenting relationship at 12-weeks postpartum at p < 0.05 were mothers’ antenatal coparenting relationship, partners’ antenatal depressive symptoms, and mothers’ perceived antenatal partner support (see **Supplementary Table 3**) and postpartum variables that individually, significantly predicted mothers’ postpartum coparenting relationship at 12-weeks postpartum were partner’s postpartum coparenting relationship, mothers’ postpartum relationship satisfaction, mothers’ postpartum anxiety symptoms at 12-weeks, mothers’ postpartum depressive symptoms at 12-weeks, and mothers’ postpartum partner support at 12-weeks (see **Supplementary Table 4**). However, when entered in the multivariable regression model, only three variables were simultaneous significant predictors of mothers’ postpartum coparenting relationship; mothers’ postpartum relationship satisfaction, mothers’ postpartum partner support, and partner’s antenatal depressive symptoms (see **Table 2**). In interpreting the model, it is interesting to note that the mothers’ postpartum partner support and mothers’ postpartum relationship satisfaction are highly correlated (0.5), so as these increase there will be a large increase in the mothers’ postpartum coparenting relationship at 12-weeks (see **Supplementary Figure 2**). Also, their coefficients were similar (mothers’ postpartum relationship satisfaction= 0.40 and mothers’ postpartum partner support = 0.47), indicating that when a mother has a high value of both of these variables, she will have a high value of the postpartum coparenting relationship. Higher scores of partner support indicate higher perceived partner support, and higher scores of relationship satisfaction indicate high relationship satisfaction.

**Table 2:** Multiple regression model predicting mothers’ postpartum coparenting relationship.

95% CI							
Variables	Coefficient	SE	Lower	Upper	VIF	t-value	p-value
(Intercept)	71.3	16.4	39.16	103.3		4.343	< 0.001
Partners’ Postpartum Relationship Satisfaction	0.36	0.15	0.07	0.65	1.30	2.34	0.021
Mothers’ Postpartum Coparenting Relationship	0.22	0.08	0.06	0.38	1.05	2.66	0.009
Partners’ Antenatal Coparenting Relationship	0.15	0.05	0.05	0.25	1.11	3.30	0.001
Partners’ Postpartum Anxiety at 12-weeks postpartum	-0.35	0.12	-0.59	-0.11	1.39	-2.87	0.005
Planned Pregnancy	6.00	2.99	0.14	11.9	1.01	2.01	0.048

SE Residual standard error: 10.02 on 94 degrees of freedom (1 observations deleted due to missingness); Multiple R-squared: 0.3177, Adjusted R-squared: 0.282; F-statistic: 8.755 on 5 and 94 DF, p-value: 7.57e-07; CI, confidence Interval; VIF, Variance Inflation Factors.

As the mothers’ postpartum relationship satisfaction and postpartum partner support worked together to predict higher scores on the postpartum relationship satisfaction, the partner’s antenatal depressive symptoms had a negative effect, with the coefficient is -0.61 (see **Table 2**). For partners it has been suggested that a score of 4 or higher may be used to screen for probable depressive symptoms, where as a score of 9 or higher may be optimal for identifying depressive symptoms (39). In our study, the mean antenatal depressive symptoms for the partners was 5.4 [Standard Deviation (SD) 4.7], suggesting that approximately 50% of the male partners had scores greater than 5 and approximately 17% had scores greater than 10, suggesting that many of the partners exhibited signs of depressive symptoms.

### 3.3 Partners

Individual regression models were examined for demographic, antenatal, and postpartum variables predicting partners’ postpartum coparenting relationship at 12-weeks postpartum (see **Supplementary Tables 5**-**7**). None of the demographic variables significantly predicted partners’ postpartum coparenting relationship at 12-weeks at the 5% level of significance (see **Supplementary Table 5**). The only significant antenatal variable was mothers’ antenatal perceived partner support (see **Supplementary Table 6**). Postpartum variables that were significant predictors of partners’ postpartum coparenting relationship at 12-weeks were mothers’ postpartum coparenting relationship, mothers’ postpartum relationship satisfaction, partners’ postpartum relationship satisfaction, both mothers’ and partners’ postpartum anxiety at 12-weeks, mothers’ postpartum partner support, partners’ postpartum depressive symptoms at 12-weeks, and partners postpartum partner support at 12-weeks (see **Supplementary Table 7**).

When included simultaneously in the multivariable regression model (see **Table 3**), mothers’ postpartum coparenting relationship, partners’ antenatal coparenting relationship, and partners’ postpartum relationship satisfaction were positive predictors of partners’ postpartum coparenting relationship at 12-weeks postpartum. Partners’ postpartum anxiety symptoms at 12-weeks were a negative predictor. In addition, planned pregnancy was identified as a confounding factor, where partners’ postpartum coparenting relationship was six points higher when the pregnancy was planned. However, only four pregnancies were unplanned. The correlations among predictor variables showed a large negative correlation between partners’ postpartum relationship satisfaction and partner’s postpartum anxiety symptoms at 12-weeks (see **Supplementary Table 8**). However, to fully interpret the effects of these variables, correlations among them must be considered (see **Supplementary Table 9**). These correlation showed that the only large correlation was between partners’ postpartum relationship satisfaction and partner postpartum anxiety symptoms at 12-weeks, which operated in opposite directions. The relationship between these variables is illustrated in **Supplementary Figure 3**.

**Table 3:** Multivariable regression model predicting partners’ postpartum coparenting relationship.

95% CI							
Variables	Coefficient	SE	Lower	Upper	VIF	t-value	p-value
(Intercept)	71.3	16.4	39.16	103.3		4.343	< 0.001
Partners’ Postpartum Relationship Satisfaction	0.36	0.15	0.07	0.65	1.30	2.34	0.021
Mothers’ Postpartum Coparenting Relationship	0.22	0.08	0.06	0.38	1.05	2.66	0.009
Partners’ Antenatal Coparenting Relationship	0.15	0.05	0.05	0.25	1.11	3.30	0.001
Partners’ Postpartum Anxiety at 12-weeks postpartum	-0.35	0.12	-0.59	-0.11	1.39	-2.87	0.005
Planned Pregnancy	6.00	2.99	0.14	11.9	1.01	2.01	0.048

SE Residual standard error: 10.07 on 92 degrees of freedom (2 observations deleted due to missingness); Multiple R-squared: 0.3259, Adjusted R-squared: 0.282; F-statistic: 7.414 on 6 and 92 DF, p-value: 1.726e-06Residual standard error: 9.612 on 87 degrees of freedom (1 observation deleted due to missingness); Multiple R-squared: 0.4189, Adjusted R-squared: 0.3388; F-statistic: 5.227 on 12 and 87 DF, p-value: 1.524e-06; CI, confidence Interval; VIF, Variance Inflation Factors.

## 4 Discussion

This study aimed to examine predictors of the postpartum coparenting relationship separately for mothers and partners among primiparous Pakistani couples in Karachi, Pakistan. To our knowledge, this is the first study conducted in LMIC to comprehensively examine the influence of parental mental health, relationship satisfaction, partner support, and antenatal coparenting relationship on postpartum coparenting relationship among both mothers and partners.

This study contributes valuable evidence by examining multiple interpersonal and psychological predictors of coparenting relationship within the sociocultural context of an LMIC. For mothers, three variables significantly predicted the postpartum coparenting relationship: mothers’ postpartum relationship satisfaction, mothers’ postpartum partner support, and partners’ antenatal depressive symptoms. For partners, significant predictors of the postpartum coparenting relationship included mothers’ postpartum coparenting relationship, partners’ antenatal coparenting relationship, partners’ postpartum relationship satisfaction, and partners’ postpartum anxiety symptoms at 12-weeks postpartum.

Relationship satisfaction was identified as a significant predictor of the postpartum coparenting relationship for both mothers and partners indicating that when spouses experience trust, emotional intimacy, and relational stability, collaborative coparenting is more likely to occur. This finding aligns with prior research demonstrating a positive association between marital relationship satisfaction and coparenting quality (40–44). Higher levels of marital satisfaction have been associated with more cooperative, supportive, and coordinated coparenting interactions (40, 44), whereas lower relationship satisfaction has been associated with conflict and may undermine coparenting quality (40). These findings are consistent with the ecological model of coparenting relationship (14) and broader family systems theories (45), which suggest that coparenting does not operate in isolation but is embedded within the broader interparental relationship. These findings underscore the interdependence between the marital and coparenting subsystems during the transition to parenthood. Future research is warranted to examine whether the quality and length of relationship prior to childbirth influences coparenting relationship during the postpartum period.

Partners’ mental health and well-being were also identified as a significant contributor to the postpartum coparenting relationship. Specifically, partners’ postnatal anxiety symptoms were inversely associated with their own postnatal coparenting quality, whereas partners’ antenatal depressive symptoms were inversely associated with mothers’ postnatal coparenting quality. These findings suggest both actor and partner effects of psychological distress on coparenting dynamics. This is consistent with previous research indicating that partners’ mental health is associated with their coparenting quality, with a higher level of psychological distress linked to lower levels of cooperative coparenting (46–48). Psychological distress may be associated with a parent’s reduced capacity to engage in shared decision-making and problem-solving (49), and may be linked with broader relational strain (50), as couples navigate the functional and emotional challenges associated with one partner’s distress (48). Additionally, elevated anxiety or depressive symptoms may be linked with heightened concerns about child well-being (51, 52), which may potentially contribute to childrearing disagreements and coparental conflict (53). Future research should examine whether relationship satisfaction may mediate the association between parental mental health and coparenting quality, particularly during the transition to parenthood.

Our findings suggest an association between antenatal coparenting relationship and postpartum coparenting relationship among partners, which is consistent with prior evidence indicating that partners’ antenatal coparenting representations are associated with postnatal coparenting quality (24). Several reasons may account for this association. First, partners who are actively involved during pregnancy by providing financial and practical support including attending prenatal clinic visits and childbirth, asking questions related to pregnancy from health care providers, and participating in prenatal activities such as ultrasound appointments are more likely to remain engaged in childrearing tasks (54) that relate to the coparenting relationship. Second, partners’ gender role beliefs may play a role in shaping coparenting relationship and partners postpartum mental health (55). Partners who endorse traditional gender norms that assign men primarily to the provider role and women to caregiving responsibilities may be less likely to engage in shared caregiving activities (56). Third, partners’ childhood experiences of family relationship may influence how they conceptualize shared parenting roles and relational cooperation after birth (57). These findings warrant further research to examine how partners’ childhood family experiences, gender role beliefs, and behavioral engagement during pregnancy interact to shape the development of coparenting after birth. Longitudinal and cross-cultural studies are particularly needed to clarify the pathways through which these factors influence postnatal coparenting quality and to inform targeted interventions for expectant partners and couples.

Our findings indicate that mothers’ postpartum coparenting relationship is associated with partners’ postpartum coparenting relationship, and mothers perceived antenatal partner support is associated with their own postpartum coparenting relationship. This is consistent with prior research that has shown that higher levels of maternal support within coparenting are associated with greater partner involvement in coparenting-related tasks (58, 59). However, some studies have suggested that maternal encouragement rather than overall coparenting quality alone is positively associated with greater partner involvement in child-related tasks (60). This divergence may reflect the mediating role of maternal encouragement, which has been shown to link maternal coparenting quality to partner support and involvement. These findings align with the concept of maternal gatekeeping (61), which describes a range of beliefs and behaviors that may either inhibit or promote collaborative parenting efforts (62), such as assuming primary responsibility for childrearing tasks, or expressing criticism of partners’ parenting efforts (60). In contrast, mothers encouragement and openness toward partners’ involvement, have been associated with greater partner participation and increased engagement in parenting activities (61) and in turn, may relate to higher perceived maternal coparenting quality. Collectively, these findings suggest that maternal behaviors may play a central mediating role in shaping partner involvement and overall coparenting quality during the postpartum period. Future research should examine the role of maternal gatekeeping in the association between mother and partner coparenting relationship within diverse sociocultural contexts.

Broadly, these findings demonstrate the importance of the coparenting relationship as a necessary element for the well-being of both parents. Evidence suggests that parental depressive symptoms are associated with poorer coparenting perceptions among both mothers and fathers, and that these associations can persist over time (63). This study further added that the negative influence of depressive symptoms on coparenting may be stronger for fathers (64) during the early parenting period, highlighting the importance of considering paternal mental health alongside maternal mental health. Our findings are consistent with this perspective, emphasizing that the coparenting relationship is closely linked to both mothers’ and partners’ psychological well-being (63). Therefore, understanding how the coparenting relationship is related to mothers’ and partners’ depressive symptoms and anxiety symptoms provides a foundation for the developing of family-centred interventions. Examining these associations separately for mothers and partners also offers valuable insight into the distinct factors that may contribute to stronger coparenting relationship and improved parental well-being. Collectively, these findings contribute to the growing body of evidence on coparenting and parental mental health, particularly in LMICs, where research on the dyadic nature of the transition to parenthood remains limited.

Future research could benefit from including more diverse samples from different regions of Pakistan to improve the generalizability of the findings. In addition, longitudinal studies that assess coparenting across multiple stages of the transition to parenthood, including the preconception period, pregnancy, and the postpartum period, would provide a more comprehensive understanding of its development over time. Furthermore, incorporating observational methods and qualitative interviews could offer richer insights into coparenting experiences and reduce reliance on self-report data. Future studies should also examine the influence of sociocultural factors on coparenting and evaluate culturally appropriate interventions that promote collaborative parenting practices in Pakistan.

### 4.1 Strengths and limitations

This study has several strengths. First, conducting research in Pakistan is a significant strength, as the general population has limited experience with research participation and research awareness. Therefore, the study provides valuable insights from an underrepresented population and contributes to the limited evidence available in this context. Second, the study addresses an important and relatively underexplored topic in Pakistan, and its findings may inform the development of relevant policies and interventions. Additionally, the inclusion of partners in this study related to coparenting is important given the patriarchal nature of society and the traditionally gendered distribution of parenting responsibilities. Furthermore, the study employed validated and reliable survey instruments, which enhanced the accuracy of the findings. Moreover, pilot testing of the tools was conducted prior to data collection to ensure clarity, cultural appropriateness, and the feasibility of the study procedures and questionnaires.

This study has several limitations. The recruitment of primiparous mothers during early pregnancy and their partners, with both partners required to consent to participation and remain in the study through 12-weeks postpartum, may have preferentially attracted couples with relatively high socioeconomic status (SES). For example, all parents in our study completed the survey in English, had at least a university-level education, and the partners were employed in stable positions within the government or private sectors. These characteristics resulted in a relatively homogeneous sample. At the same time, these eligibility criteria, combined with the fact that relatively few pregnant women attend prenatal clinics with their partners, restricted the pool of eligible participants. For example, enrolling 101 couples during early pregnancy, with both partners consenting to remain in the study through 12-weeks postpartum, required access to a large population of pregnant women. Consequently, the relatively modest sample size should be interpreted from this context. The relatively small sample size with participant attrition may have reduced statistical power, introduced attrition bias, and limited the representativeness of the findings. Second, participants were primiparous couples recruited from a single tertiary care hospital in urban Pakistan and were limited to English speaking couples. These characteristics may limit the generalizability of the findings to couples with multiple parities, those receiving care in rural healthcare settings, and populations with different linguistic, cultural, and sociodemographic characteristics. In particular, recruitment from a single tertiary care hospital may have resulted in a sample with characteristics that differ from couples receiving antenatal care in other healthcare settings or regions of Pakistan. Therefore, the findings should be interpreted within the context of the study population and setting and may not be generalizable to the broader Pakistani population or to populations in other countries. Third, the use of self-reported measures may introduce reporting and social desirability bias, although confidentiality and anonymity were emphasized to minimize this risk. Common method bias is also possible because the same participants completed the survey instruments; however, the variables were assessed at different time points, which may have reduced this bias. In addition, the parents who participated may represent a more supportive and engaged subset, as both parents were present in the clinic at the time of recruitment and consented to take part in the study, suggesting that the present findings may be more reflective of highly engaged individuals than of the wider population of expectant parents in Pakistan. Finally, the sociocultural context of Pakistan, where traditional gender roles influence parenting responsibilities, should be considered when interpreting the findings and their applicability to other settings.

### 4.2 Implications

This study’s findings highlight key implications, including the importance of developing healthy coparenting relationship among first-time parents. Institutional antenatal and postnatal awareness programs must focus on healthy relationships and partner support. Improving the quality of coparenting will strengthen family functioning during the transition to parenthood. The findings also suggest that the mental well-being of male partners is extremely important from the baby’s conception through delivery. New parents often face high levels of anxiety and depressive symptoms due to a lack of knowledge, skills, and capacity for childbearing, limited access to relevant information, and few training opportunities. Timely screening for anxiety and depressive symptoms is important, and couples should be trained to ensure that experiencing such anxiety and depressive symptoms is normal and that they can reach out to healthcare providers for professional help. Routine screening for partner anxiety and depressive symptoms during pregnancy and postpartum could help identify families at risk for coparenting difficulties. Access to mental health support should be available for couples in Pakistan, and providing mental health resources and supportive parenting programs such as coparenting intervention may enhance partner engagement and overall family functioning. In LMICs, less emphasis is given to marital counselling, and minor conflicts can escalate into major family disputes. Findings suggest that marital counselling is a key intervention for strengthening the marital relationship and promoting positive coparenting. While individual mental health counselling facilities are available in LMICs, including Pakistan, couples counselling for new or expectant parents is not commonly introduced. The study also highlights that the coparenting process should begin before childbirth, when the couple is planning to have a baby. In some cultures, in Pakistan, partners often need more encouragement and motivation to participate in prenatal care and childbirth. Partners’ early involvement may increase their responsibility for positive coparenting after the baby’s birth.

### 4.3 Conclusion

This study identified several variables associated with postpartum coparenting relationship among mothers and partners during the transition to parenthood. For mothers, the postpartum coparenting relationship was associated with postpartum relationship satisfaction, postpartum partner support, and partners’ antenatal depressive symptoms. For partners, postpartum coparenting relationship was associated with mothers’ postpartum coparenting relationship, partners’ antenatal coparenting relationship, partners’ postpartum relationship satisfaction, and partners’ postpartum anxiety symptoms at 12-weeks postpartum. These findings highlight the interconnected nature of parental relationship dynamics and psychological well-being during the postpartum period. Although enrolling only primiparous mothers at early pregnancy and their partners who completed follow-up through 12-weeks postpartum restricted the target population size, this approach ensured a homogenous sample, enabling precise parameter estimation producing more reliable model fit than is possible with heterogenous samples of similar size. Additionally, coparenting relationship was assessed only during pregnancy and at 12-weeks postpartum. Future research should examine coparenting relationship and related factors across multiple time points using longitudinal study designs to better understand how these relationships evolve over time and to identify potential long-term predictors and outcomes. This study emphasizes the importance of considering both maternal and partner factors when examining coparenting relationship in early parenthood. Understanding these factors may help inform the development of targeted interventions aimed at strengthening the coparenting relationship and promoting family well-being during the transition to parenthood.

## Data Availability

The raw data supporting the conclusions of this article will be made available by the authors, without undue reservation.
