# Supplementary material for: Predictors of postpartum coparenting relationship among primiparous couples in Karachi, Pakistan
Source: Front Psychiatry. 2026 Sep 25;17:1886830. doi: 10.3389/fpsyt.2026.1886830 (PMC13651462; doi:10.3389/fpsyt.2026.1886830)
Supplement: Supplementary file 1 [file Supplementaryfile1.docx]

**Supplementary Material**

1. **Supplementary Figures**

**Supplementary Figure 1:** STROBE Diagram of Participant Flow

Note: RDAS= Revised Dyadic Attachment Scale to measure relationship satisfaction; PPSS Postpartum Partner Support to measure partner support

**Supplementary Figure 2**: Relationship between the Mothers’ Postpartum Relationship Satisfaction and Mothers’ Postpartum Partner Support

Note: STAI State -Trait Anxiety Inventory to measure anxiety; RDAS Revised Dyadic Attachment Scale to measure relationship satisfaction

**Supplementary Figure 3:** Relationship between Partner Postpartum Relationship Satisfaction and 1Partners’ Postpartum Anxiety at 12-weeks

2. **Supplementary Tables**
   2.1. **Supplementary Table 1:**

Correlations between Variables

|  | Mothers’ Age | Years of Marriage | Weeks of Pregnancy | MACR | PACR | MARS | PARS | MAPHQ | PAPHQ | MAPS |
| --- | --- | --- | --- | --- | --- | --- | --- | --- | --- | --- |
| Partner Age | 0.298 | 0.127 | -0.071 | -0.18 | -0.081 | 0.038 | 0.108 | 0.055 | -0.084 | -0.11 |
| Mother Age | 1 | 0.227 | -0.106 | -0.022 | -0.003 | -0.055 | -0.076 | -0.147 | 0.022 | -0.159 |
| Years of Marriage |  | 1 | -0.094 | -0.002 | 0.073 | 0.011 | -0.077 | -0.096 | -0.002 | -0.034 |
| Weeks of Pregnancy |  |  | 1 | -0.022 | -0.034 | -0.055 | -0.002 | -0.142 | 0.06 | -0.152 |
| MACR |  |  |  | 1 | -0.035 | 0.236 | 0.172 | -0.017 | -0.041 | 0.464 |
| PACR |  |  |  |  | 1 | -0.28 | -0.236 | 0.125 | 0.137 | -0.1 |
| MARS |  |  |  |  |  | 1 | 0.644 | -0.297 | -0.159 | 0.539 |
| PARS |  |  |  |  |  |  | 1 | -0.279 | -0.317 | 0.27 |
| MAPHQ |  |  |  |  |  |  |  | 1 | 0.248 | -0.107 |
| PAPHQ |  |  |  |  |  |  |  |  | 1 | -0.149 |
| MAPS |  |  |  |  |  |  |  |  |  | 1 |

Note: MACR Mothers’ Antenatal Coparenting Relationship; PACR Partners’ Antenatal Coparenting Relationship; MARS Mothers’ Antenatal Relationship Satisfaction; PARS Partners’ Antenatal Relationship Satisfaction; MAPHQ Mothers Antenatal Depressive Symptoms using PHQ; PAPHQ Partners’ Antenatal Depressive Symptoms using PHQ; MAPS Mothers; Antenatal Partner Support

**2.1. Supplementary Table 1 (cont’d)**

|  | PAPS | MAA | PAA | MAEPDS | PAEPDS | MFU1A | PFU1A | MFU1D | PFU1D | MPCR |
| --- | --- | --- | --- | --- | --- | --- | --- | --- | --- | --- |
| Partner Age | 0.024 | 0.063 | -0.24 | 0.029 | -0.297 | -0.018 | -0.092 | -0.042 | -0.225 | 0.086 |
| Mother Age | -0.005 | 0.001 | 0.013 | -0.13 | -0.109 | -0.017 | -0.096 | -0.125 | -0.181 | 0.066 |
| Years of Marriage | -0.109 | -0.1 | -0.103 | -0.16 | 0.111 | -0.098 | -0.092 | -0.188 | -0.087 | -0.124 |
| Weeks of Pregnancy | 0.022 | -0.231 | 0.074 | -0.016 | 0.048 | -0.074 | -0.012 | -0.051 | 0.017 | -0.076 |
| MACR | 0.166 | -0.086 | -0.1 | -0.195 | -0.063 | -0.241 | -0.188 | -0.158 | -0.114 | 0.252 |
| PACR | -0.089 | 0.024 | 0.081 | 0.141 | 0.177 | 0.04 | 0.167 | 0.049 | 0.366 | -0.176 |
| MARS | 0.323 | -0.295 | -0.277 | -0.24 | -0.209 | -0.31 | -0.265 | -0.379 | -0.276 | 0.16 |
| PARS | 0.489 | -0.19 | -0.411 | -0.151 | -0.331 | -0.358 | -0.334 | -0.345 | -0.335 | 0.071 |
| MAPHQ | -0.237 | 0.633 | 0.195 | 0.063 | 0.205 | 0.309 | 0.141 | 0.398 | 0.207 | 0.053 |
| PAPHQ | -0.348 | 0.178 | 0.605 | 0.065 | 0.464 | 0.176 | 0.291 | 0.142 | 0.342 | -0.09 |
| MAPS | 0.139 | -0.191 | -0.24 | -0.183 | -0.208 | -0.211 | -0.2 | -0.214 | -0.235 | 0.296 |
| PAPS | 1 | -0.156 | -0.379 | -0.03 | -0.333 | -0.254 | -0.278 | -0.254 | -0.329 | 0.011 |
| MAA |  | 1 | 0.22 | 0.147 | 0.23 | 0.356 | -0.006 | 0.381 | 0 | 0.031 |
| PAA |  |  | 1 | 0.186 | 0.62 | 0.142 | 0.394 | 0.203 | 0.316 | -0.187 |
| MAEPDS |  |  |  | 1 | 0.108 | 0.211 | 0.243 | 0.274 | 0.117 | -0.158 |
| PAEPDS |  |  |  |  | 1 | 0.177 | 0.302 | 0.224 | 0.362 | -0.28 |
| MFU1A |  |  |  |  |  | 1 | 0.329 | 0.607 | 0.22 | -0.092 |
| PFU1A |  |  |  |  |  |  | 1 | 0.307 | 0.65 | -0.034 |
| MFU1D |  |  |  |  |  |  |  | 1 | 0.381 | -0.094 |
| PFU1D |  |  |  |  |  |  |  |  | 1 | -0.029 |

Note: MACR Mothers’ Antenatal Coparenting Relationship; PACR Partners’ Antenatal Coparenting Relationship; MARS Mothers’ Antenatal Relationship Satisfaction; PARS Partners’ Antenatal Relationship Satisfaction; MAPHQ Mothers Antenatal Depressive Symptoms using PHQ; PAPHQ Partners’ Antenatal Depressive Symptoms using PHQ; MAPS Mothers; Antenatal Partner Support; PAPS Partners’ Antenatal Partner Support; MAA Mothers’ Antenatal Anxiety Symptoms; PAA Partners’ Antenatal Anxiety Symptoms; MAEPDS Mothers Antenatal Depressive Symptoms using EPDS; PAEPDS Partners’ Antenatal Depressive Symptoms using EPDS; MFU1A Mothers Postpartum Anxiety Symptoms at 4-6-weeks; PFU1A Partners’ Postpartum Anxiety Symptoms at 4-6-weeks; MFU1D Mothers’ Postpartum Depressive Symptoms using EPDS at 4-6-weeks; PFU1D Partners’ Postpartum Depressive Symptoms using EPDS at 4-6-weeks

**2.1. Supplementary Table 1 (cont’d)**

|  | PPCR | MPRS | PPRS | MPD | PPD | MPA | PPA | MPPS | PPSS |
| --- | --- | --- | --- | --- | --- | --- | --- | --- | --- |
| Partner Age | 0.097 | -0.032 | 0.024 | -0.03 | -0.074 | -0.03 | -0.138 | -0.146 | 0.059 |
| Mother Age | -0.04 | 0.085 | -0.091 | -0.079 | 0.017 | 0.014 | 0.085 | 0.005 | -0.18 |
| Years of Marriage | -0.028 | -0.084 | -0.137 | -0.179 | 0.105 | -0.118 | -0.006 | -0.06 | -0.087 |
| Weeks of Pregnancy | 0.027 | -0.044 | 0.003 | -0.021 | 0.029 | -0.052 | -0.087 | -0.133 | 0.047 |
| MACR | -0.004 | 0.325 | 0.046 | -0.264 | -0.149 | -0.172 | -0.053 | 0.185 | 0.255 |
| PACR | 0.151 | -0.145 | -0.082 | 0.146 | 0.221 | -0.005 | 0.277 | -0.051 | -0.023 |
| MARS | 0.164 | 0.475 | 0.238 | -0.262 | -0.182 | -0.133 | -0.267 | 0.154 | 0.202 |
| PARS | 0.083 | 0.344 | 0.285 | -0.279 | -0.281 | -0.085 | -0.292 | 0.115 | 0.213 |
| MAPHQ | -0.035 | -0.101 | 0.006 | 0.316 | 0.068 | 0.165 | 0.136 | 0.069 | 0.23 |
| PAPHQ | 0.02 | 0.041 | -0.084 | 0.202 | 0.257 | 0.076 | 0.215 | 0.035 | 0.085 |
| MAPS | 0.238 | 0.412 | 0.212 | -0.23 | -0.206 | -0.18 | -0.277 | 0.373 | 0.246 |
| PAPS | 0.014 | 0.117 | 0.125 | -0.191 | -0.265 | -0.031 | -0.215 | -0.029 | 0.044 |
| MAA | -0.11 | -0.088 | -0.164 | 0.319 | 0.087 | 0.27 | 0.17 | 0.024 | 0.102 |
| PAA | -0.033 | -0.074 | -0.222 | 0.258 | 0.231 | 0.181 | 0.342 | 0.001 | 0.052 |
| MAEPDS | -0.056 | -0.212 | -0.057 | 0.382 | 0.12 | 0.078 | 0.132 | -0.15 | -0.048 |
| PAEPDS | -0.104 | -0.221 | -0.316 | 0.269 | 0.388 | 0.197 | 0.36 | -0.128 | -0.077 |
| MFU1A | -0.115 | -0.135 | -0.172 | 0.497 | 0.095 | 0.46 | 0.301 | 0.023 | 0.07 |
| PFU1A | -0.049 | -0.174 | -0.228 | 0.331 | 0.48 | 0.148 | 0.448 | 0.027 | -0.019 |
| MFU1D | -0.131 | -0.164 | -0.192 | 0.588 | 0.306 | 0.372 | 0.342 | -0.031 | -0.056 |
| PFU1D | -0.035 | -0.257 | -0.198 | 0.277 | 0.542 | 0.165 | 0.457 | 0.029 | -0.024 |
| MPCR | 0.244 | 0.452 | 0.125 | -0.281 | -0.1 | -0.324 | -0.137 | 0.489 | 0.097 |
| PPCR | 1 | 0.261 | 0.367 | -0.144 | -0.273 | -0.279 | -0.356 | 0.231 | 0.238 |
| MPRS |  | 1 | 0.366 | -0.242 | -0.194 | -0.391 | -0.144 | 0.499 | 0.445 |
| PPRS |  |  | 1 | -0.315 | -0.357 | -0.364 | -0.465 | 0.168 | 0.377 |
| MPD |  |  |  | 1 | 0.39 | 0.594 | 0.452 | -0.17 | -0.091 |
| PPD |  |  |  |  | 1 | 0.232 | 0.689 | -0.106 | -0.235 |
| MPA |  |  |  |  |  | 1 | 0.472 | -0.256 | -0.319 |
| PPA |  |  |  |  |  |  | 1 | -0.112 | -0.236 |
| MPPS |  |  |  |  |  |  |  | 1 | 0.306 |
| PPPS |  |  |  |  |  |  |  |  | 1 |

Note: MACR Mothers’ Antenatal Coparenting Relationship; PACR Partners’ Antenatal Coparenting Relationship; MARS Mothers’ Antenatal Relationship Satisfaction ; PARS Partners’ Antenatal Relationship Satisfaction ; MAPHQ Mothers Antenatal Depressive symptoms using PHQ; PAPHQ Partners’ Antenatal Depressive symptoms using PHQ ; MAPS Mothers Antenatal Perceived Partner Support; PAPS Partners’ Antenatal Perceived Partner Support; MAA Mothers’ Antenatal Anxiety symptoms; PAA Partners’ Antenatal Anxiety symptoms; MAEPDS Mothers’ Antenatal Depressive symptoms using EPDS; PAEPDS Partners’ Antenatal Depressive symptoms using EPDS; MFU1A Mothers Postpartum Anxiety Symptoms at 4-6-weeks; PFU1A Partners’ Postpartum Anxiety symptoms at 4-6-weeks; MFU1D Mothers’ Postpartum Depressive symptoms at 4-6-weeks; PFU1D Partners’ Postpartum Depressive Symptoms at 4-6-weeks; MPCR Mothers’ Postpartum Coparenting Relationship; PPCR Partners’ Postpartum Coparenting Relationship; MPRS Mothers’ Postpartum Relationship Satisfaction; PPRS Partners’ Postpartum Relationship Satisfaction; MPD Mothers’ Postpartum Depressive Symptoms at 12-weeks; PPD Partners’ Postpartum Depressive Symptoms at 12-weeks; MPA Mothers Postpartum Anxiety Symptoms at 12-weeks; PPA Partners’ Postpartum Anxiety Symptoms at 12-weeks; MPPS Mothers’ Postpartum Partner Support; PPSS Partners’ Postpartum Partner Support

2.2. **Supplementary Table 2**

Summary of Individual Regression Models: Demographic Variables Predicting Mother Postpartum Coparenting Relationship

|  | Estimate | Std. Error | t value | p-value | Correlation |
| --- | --- | --- | --- | --- | --- |
| (Intercept) | 123.52 | 10.74 | 11.50 | <0.001 |  |
| Mothers Age | 0.26 | 0.40 | 0.66 | 0.509 | 0.0664 |
| (Intercept) | 121.50 | 11.20 | 10.85 | <0.001 |  |
| Partners Age | 0.31 | 0.36 | 0.85 | 0.397 | 0.0857 |
| (Intercept) | 132.27 | 1.84 | 71.86 | <0.001 |  |
| Years of Marriage | -0.09 | 0.07 | -1.25 | 0.215 | 0.1244 |
| (Intercept) | 133.66 | 4.23 | 31.59 | <0.001 |  |
| Weeks Pregnant | -0.19 | 0.24 | -0.76 | 0.448 | 0.0764 |
| (Intercept) | 127.43 | 4.78 | 26.63 | **<0.001** |  |
| Mothers Education: Bachelors | 3.60 | 5.05 | 0.71 |  |  |
| Mothers Education: Graduate (Master’s and PhD) | 3.00 | 5.27 | 0.57 |  |  |
| (Intercept) | 127.75 | 6.34 | 20.16 | **<0.001** |  |
| Partners Education: Bachelors | 2.65 | 6.60 | 0.40 |  |  |
| Partners Education: Graduate (Master’s and PhD) | 3.23 | 6.59 | 0.49 |  |  |
| (Intercept) | 121.33 | 5.12 | 23.68 | **<0.001** |  |
| Mother Ethnicity: Sindhi | 7.30 | 6.37 | 1.15 |  |  |
| Mother Ethnicity: Katchi | 17.67 | 7.60 | 2.32 |  |  |
| Mother Ethnicity: Gujrati | 11.22 | 6.62 | 1.70 |  |  |
| Mother Ethnicity: Punjabi | 9.60 | 6.06 | 1.58 |  |  |
| Mother Ethnicity: Pathan | 6.33 | 8.88 | 0.71 |  |  |
| Mother Ethnicity: Others | 12.00 | 5.92 | 2.03 |  |  |
| Mother Ethnicity: Urdu | 8.40 | 5.56 | 1.51 |  |  |
| (Intercept) | 128.25 | 6.41 | 20.00 | **<0.001** |  |
| Partner Ethnicity: Sindhi | 1.06 | 7.17 | 0.15 |  |  |
| Partner Ethnicity: Katchi | 12.00 | 9.07 | 1.32 |  |  |
| Partner Ethnicity: Gujrati | 3.00 | 7.40 | 0.41 |  |  |
| Partner Ethnicity: Punjabi | 2.63 | 7.13 | 0.37 |  |  |
| Partner Ethnicity: Pathan | -5.25 | 14.34 | -0.37 |  |  |
| Partner Ethnicity: Others | 1.44 | 7.33 | 0.20 |  |  |
| Partner Ethnicity: Urdu | 2.25 | 6.78 | 0.33 |  |  |
| (Intercept) | 129.67 | 1.55 | 83.85 | <0.001 |  |
| Marriage Type | 2.65 | 2.63 | 1.01 | 0.316 |  |
| (Intercept) | 130.60 | 1.35 | 96.47 | <0.001 |  |
| Planned Pregnancy | -0.10 | 3.64 | -0.03 | 0.979 |  |
| (Intercept) | 130.43 | 1.84 | 70.81 | <0.001 |  |
| Family Type | 0.30 | 2.52 | 0.12 | 0.906 |  |
| (Intercept) | 131.27 | 1.41 | 93.33 | <0.001 |  |
| Birth Outcomes | -4.70 | 3.68 | -1.28 | 0.205 |  |

2.3. **Supplementary Table 3**

Summary of Individual regression Models: Antenatal Variables predicting Mother Postpartum Coparenting Relationship at 12-weeks postpartum

|  | **Estimate** | **Std. Error** | **t value** | **p-value** | **Correlation** |
| --- | --- | --- | --- | --- | --- |
| (Intercept) | 94.427 | 13.988 | 6.751 | <0.001 |  |
| Mothers’ Antenatal Coparenting Relationship | 0.276 | 0.107 | 2.595 | **0.011** | 0.252 |
| (Intercept) | 142.154 | 6.608 | 21.511 | <0.001 |  |
| Partners’ Antenatal Coparenting Relationship | -0.098 | 0.055 | -1.782 | 0.078 | 0.176 |
| (Intercept) | 116.483 | 8.845 | 13.170 | <0.001 |  |
| Mothers’ Antenatal Relationship Satisfaction | 0.252 | 0.157 | 1.610 | 0.111 | 0.160 |
| (Intercept) | 124.243 | 9.089 | 13.669 | <0.001 |  |
| Partners’ Antenatal Relationship Satisfaction | 0.116 | 0.165 | 0.704 | 0.483 | 0.071 |
| (Intercept) | 135.181 | 3.139 | 43.071 | <0.001 |  |
| Mothers’ Antenatal Depressive Symptoms | -0.580 | 0.364 | -1.594 | 0.114 | 0.158 |
| (Intercept) | 135.862 | 2.183 | 62.236 | <0.001 |  |
| Partners’ Antenatal Depressive Symptoms | -0.769 | 0.265 | -2.901 | **0.005** | 0.280 |
| (Intercept) | 129.651 | 2.167 | 59.818 | <0.001 |  |
| Mothers’ Antenatal PHQ Score | 0.147 | 0.278 | 0.528 | 0.599 | 0.053 |
| (Intercept) | 131.94 | 1.96 | 67.42 | <0.001 |  |
| Partners’ Antenatal PHQ Score | -0.304 | 0.337 | -0.902 | 0.369 | -0.09 |
| (Intercept) | 129.162 | 4.755 | 27.165 | <0.001 |  |
| Mothers’ Antenatal Anxiety Symptoms | 0.043 | 0.138 | 0.310 | 0.757 | 0.031 |
| (Intercept) | 137.789 | 3.990 | 34.533 | <0.001 |  |
| Partners’ Antenatal Anxiety Symptoms | -0.224 | 0.118 | -1.899 | 0.060 | 0.187 |
| (Intercept) | 92.285 | 12.618 | 7.314 | <0.001 |  |
| Mother’s Antenatal Perceived Partner Support | 0.534 | 0.174 | 3.066 | **0.003** | 0.296 |
| (Intercept) | 129.007 | 14.205 | 9.082 | <0.001 |  |
| Partners’ Antenatal Perceived Partner Support | 0.022 | 0.195 | 0.111 | 0.911 | 0.011 |

Note: PHQ Patient Health Questionnaire

**2.4. Supplementary Table 4**

Summary of Individual regression Models: Postpartum Variables predicting Mother Postpartum Coparenting Relationship

|  | **Estimate** | **Std. Error** | **t value** | **p-value** | **Correlation** |
| --- | --- | --- | --- | --- | --- |
| (Intercept) | 133.93 | 3.97 | 33.76 | <0.001 |  |
| Mother Postpartum Anxiety Symptoms at 4-6-weeks | -0.10 | 0.11 | -0.89 | 0.374 | 0.09 |
| (Intercept) | 131.85 | 4.17 | 31.64 | <0.001 |  |
| Partner Postpartum Anxiety Symptoms at 4-6-weeks | -0.04 | 0.13 | -0.33 | 0.746 | 0.03 |
| (Intercept) | 132.84 | 2.79 | 47.65 | <0.001 |  |
| Mother Postpartum Depressive Symptoms at 4-6-weeks | -0.25 | 0.27 | -0.92 | 0.361 | 0.09 |
| (Intercept) | 131.13 | 2.31 | 56.69 | <0.001 |  |
| Partner Postpartum Depressive Symptoms at 4-6-weeks | -0.08 | 0.29 | -0.27 | 0.785 | 0.03 |
| (Intercept) | 95.68 | 14.04 | 6.81 | <0.001 |  |
| Partner Postpartum Coparenting Relationship | 0.26 | 0.10 | 2.49 | **0.014** | 0.24 |
| (Intercept) | 85.43 | 9.26 | 9.22 | <0.001 |  |
| Mother postpartum Relationship Satisfaction | 0.82 | 0.17 | 4.92 | **<0.001** | 0.45 |
| (Intercept) | 118.84 | 9.45 | 12.57 | <0.001 |  |
| Partner Postpartum Relationship Satisfaction | 0.21 | 0.17 | 1.25 | 0.213 | 0.12 |
| (Intercept) | 143.82 | 4.06 | 35.45 | <0.001 |  |
| Mother postpartum Anxiety Symptoms at 12-weeks | -0.40 | 0.12 | -3.41 | **0.001** | 0.32 |
| (Intercept) | 136.00 | 4.14 | 32.88 | <0.001 |  |
| Partner Postpartum Anxiety Symptoms at 12-weeks | -0.18 | 0.13 | -1.37 | 0.173 | 0.14 |
| (Intercept) | 136.25 | 2.29 | 59.45 | <0.001 |  |
| Mother Postpartum Depressive Symptoms at 12-weeks | -0.78 | 0.27 | -2.91 | **0.004** | 0.28 |
| (Intercept) | 132.03 | 1.92 | 68.90 | <0.001 |  |
| Partner Postpartum Depressive Symptoms at 12-weeks | -0.27 | 0.27 | -1.00 | 0.322 | 0.10 |
| (Intercept) | 86.98 | 7.90 | 11.01 | <0.001 |  |
| Mother Postpartum Partner Support | 0.61 | 0.11 | 5.54 | **<0.001** | 0.49 |
| (Intercept) | 121.76 | 9.12 | 13.35 | <0.001 |  |
| Partner Postpartum Partner Support | 0.12 | 0.13 | 0.97 | 0.337 | 0.10 |

# **2.5. Supplementary Table 5**

Summary of Individual Regression Models: Demographic Variables Predicting Partners’ Postpartum Coparenting Relationship

|  | Estimate | Std. Error | t value | P value | Correlation |
| --- | --- | --- | --- | --- | --- |
| (Intercept) | 137.85 | 10.15 | 13.57 | <0.001 |  |
| Mother Age | -0.15 | 0.38 | -0.39 | 0.694 | 0.040 |
| (Intercept) | 122.93 | 11.36 | 10.82 | <0.001 |  |
| Partner Age | 0.36 | 0.37 | 0.96 | 0.337 | 0.097 |
| (Intercept) | 134.23 | 1.75 | 76.59 | <0.001 |  |
| Years of Marriage | -0.02 | 0.07 | -0.28 | 0.783 | 0.028 |
| (Intercept) | 132.83 | 4.02 | 33.08 | <0.001 |  |
| Weeks Pregnant | 0.06 | 0.23 | 0.27 | 0.787 | 0.027 |
| (Intercept) | 139.29 | 4.47 | 31.13 | <0.001 |  |
| Mothers Education: Bachelors | -5.52 | 4.73 | -1.17 | 0.433 |  |
| Mothers Education: Graduate (Master’s and PhD) | -6.38 | 4.93 | -1.29 |  |  |
| (Intercept) | 136.00 | 5.93 | 22.93 | <0.001 |  |
| Partner Education: Bachelors | -3.55 | 6.18 | -0.58 | 0.524 |  |
| Partner Education: Graduate (Master’s and PhD) | -0.94 | 6.17 | -0.15 |  |  |
| (Intercept) | 137.00 | 4.82 | 28.43 | <0.001 |  |
| Mother Ethnicity: Sindhi | -6.70 | 6.09 | -1.10 | 0.408 |  |
| Mother Ethnicity: Katchi | -1.00 | 7.15 | -0.14 |  |  |
| Mother Ethnicity: Gujrati | 0.44 | 6.22 | 0.07 |  |  |
| Mother Ethnicity: Punjabi | -6.67 | 5.70 | -1.17 |  |  |
| Mother Ethnicity: Pathan | -13.33 | 8.35 | -1.60 |  |  |
| Mother Ethnicity: Others | -4.17 | 5.56 | -0.75 |  |  |
| Mother Ethnicity: Urdu | -0.88 | 5.23 | -0.17 |  |  |
| (Intercept) | 137.50 | 5.93 | 23.20 | <0.001 |  |
| Partner Ethnicity: Sindhi | -5.50 | 6.67 | -0.82 | 0.496 |  |
| Partner Ethnicity: Katchi | 3.50 | 8.38 | 0.42 |  |  |
| Partner Ethnicity: Gujrati | -3.08 | 6.84 | -0.45 |  |  |
| Partner Ethnicity: Punjabi | -3.68 | 6.59 | -0.56 |  |  |
| Partner Ethnicity: Pathan | 9.50 | 13.25 | 0.72 |  |  |
| Partner Ethnicity: Others | -9.04 | 6.78 | -1.33 |  |  |
| Partner Ethnicity: Urdu | -2.56 | 6.27 | -0.41 |  |  |
| (Intercept) | 134.69 | 1.47 | 91.83 | <0.001 |  |
| Marriage Type | -2.35 | 2.48 | -0.95 | 0.346 |  |
| (Intercept) | 133.07 | 1.25 | 106.14 | <0.001 |  |
| Planned Pregnancy | 6.16 | 3.48 | 1.77 | 0.079 |  |
| (Intercept) | 131.72 | 1.71 | 77.16 | <0.001 |  |
| Family Type | 4.05 | 2.34 | 1.73 | 0.087 |  |
| (Intercept) | 133.17 | 1.28 | 103.70 | <0.001 |  |
| Birth Outcomes | 2.76 | 3.35 | 0.82 | 0.412 |  |

**2.6. Supplementary Table 6**

Summary of Individual Regression Models: Antenatal Variables Predicting Partner Postpartum Coparenting Relationship

|  | Estimate | Std. Error | t value | Pr(>\|t\|) | Correlation |
| --- | --- | --- | --- | --- | --- |
| (Intercept) | 134.35 | 13.79 | 9.75 | < 0.001 |  |
| Mother Antenatal Coparenting Relationship | 0.00 | 0.10 | -0.04 | 0.972 | 0.004 |
| (Intercept) | 124.57 | 6.24 | 19.95 | <0.001 |  |
| Partner Antenatal Coparenting Relationship | 0.08 | 0.05 | 1.52 | 0.133 | 0.004 |
| (Intercept) | 120.14 | 8.45 | 14.22 | <0.001 |  |
| Mother Antenatal Relationship Satisfaction | 0.24 | 0.15 | 1.64 | 0.104 | 0.151 |
| (Intercept) | 126.78 | 8.64 | 14.67 | <0.001 |  |
| Partner Antenatal Relationship Satisfaction | 0.13 | 0.16 | 0.83 | 0.409 | 0.164 |
| (Intercept) | 135.38 | 2.99 | 45.23 | <0.001 |  |
| Mother Antenatal Depressive Symptoms | -0.19 | 0.35 | -0.55 | 0.583 | 0.083 |
| (Intercept) | 135.74 | 2.16 | 62.96 | <0.001 |  |
| Partner Antenatal Depressive Symptoms | -0.27 | 0.26 | -1.04 | 0.302 | 0.056 |
| (Intercept) | 134.44 | 2.04 | 65.74 | <0.001 |  |
| Mother Antenatal PHQ Score | -0.09 | 0.26 | -0.35 | 0.731 | 0.104 |
| (Intercept) | 133.58 | 1.86 | 65.74 | <0.001 |  |
| Partner Antenatal PHQ Score | 0.06 | 0.32 | -0.35 | 0.731 | 0.035 |
| (Intercept) | 138.55 | 4.45 | 71.95 | <0.001 |  |
| Mother Antenatal Anxiety Symptoms | -0.14 | 0.13 | -0.19 | 0.842 | 0.110 |
| (Intercept) | 135.07 | 3.84 | 35.20 | <0.001 |  |
| Partner Antenatal Anxiety Symptoms | -0.04 | 0.11 | -0.33 | 0.744 | 0.033 |
| (Intercept) | 103.49 | 12.63 | 8.19 | <0.001 |  |
| Mother Antenatal Perceived Partner Support | 0.42 | 0.17 | 2.41 | **0.018** | 0.238 |
| (Intercept) | 132.01 | 13.42 | 9.84 | <0.001 |  |
| Partner Antenatal Perceived Partner Support | 0.03 | 0.18 | 0.14 | 0.889 | 0.014 |

Note: PHQ Patient Health Questionnaire

**2.7.** **Supplementary Table 7**

Summary of Individual Regression Models: Postpartum Variables Predicting Partner Postpartum Coparenting Relationship

|  | Estimate | Std. Error | t value | Pr(>\|t\|) | Correlation |
| --- | --- | --- | --- | --- | --- |
| (Intercept) | 137.33 | 3.58 | 38.41 | <0.001 |  |
| Mother Postpartum Anxiety Symptoms at 4-6-weeks | -0.11 | 0.10 | -1.11 | 0.269 | 0.115 |
| (Intercept) | 134.87 | 3.85 | 35.06 | <0.001 |  |
| Partner Postpartum Anxiety Symptoms at 4-6-weeks | -0.05 | 0.12 | -0.46 | 0.647 | 0.049 |
| (Intercept) | 136.41 | 2.51 | 54.34 | <0.001 |  |
| Mother Postpartum Depressive Symptoms at 4-6-weeks | -0.31 | 0.24 | -1.28 | 0.205 | 0.131 |
| (Intercept) | 133.74 | 2.13 | 62.65 | <0.001 |  |
| Partner Postpartum Depressive Symptoms at 4-6-weeks | -0.09 | 0.27 | -0.33 | 0.744 | 0.035 |
| (Intercept) | 104.01 | 12.04 | 8.64 | <0.001 |  |
| Mother Postpartum Coparenting Relationship | 0.23 | 0.09 | 2.49 | **0.014** | 0.244 |
| (Intercept) | 106.97 | 10.31 | 10.38 | <0.001 |  |
| Mother Postpartum Relationship Satisfaction | 0.48 | 0.18 | 2.61 | **0.011** | 0.261 |
| (Intercept) | 101.59 | 8.35 | 12.16 | <0.001 |  |
| Partner Postpartum Relationship Satisfaction | 0.59 | 0.15 | 3.90 | **<0.001** | 0.367 |
| (Intercept) | 144.61 | 3.90 | 37.04 | <0.001 |  |
| Mother Postpartum Anxiety Symptoms at 12-weeks | -0.33 | 0.11 | -2.88 | **0.005** | 0.279 |
| (Intercept) | 147.10 | 3.68 | 39.95 | <0.001 |  |
| Partner Postpartum Anxiety Symptoms at 12-weeks | -0.43 | 0.11 | -3.77 | **<0.001** | 0.356 |
| (Intercept) | 136.59 | 2.22 | 61.43 | <0.001 |  |
| Mother Postpartum Depressive Symptoms at 12-weeks | -0.38 | 0.26 | -1.44 | 0.153 | 0.144 |
| (Intercept) | 137.64 | 1.76 | 78.05 | <0.001 |  |
| Partner Postpartum Depressive Symptoms at 12-weeks | -0.69 | 0.25 | -2.81 | **0.006** | 0.273 |
| (Intercept) | 113.50 | 8.67 | 13.09 | <0.001 |  |
| Mother Postpartum Partner Support | 0.28 | 0.12 | 2.34 | **0.021** | 0.231 |
| (Intercept) | 112.93 | 8.78 | 12.86 | <0.001 |  |
| Partner Postpartum Partner Support | 0.30 | 0.12 | 2.41 | **0.018** | 0.238 |

**2.8. Supplementary Table 8**

Correlations between the predictor variables: The multivariable regression model predicting Partner Postpartum Coparenting Relationship

|  | Mother Postpartum Coparenting Relationship | Partner Postpartum Relationship Satisfaction | Partner postpartum Anxiety at12-weeks |
| --- | --- | --- | --- |
| Partner Antenatal Coparenting Relationship | -0.176 | -0.082 | 0.277 |
| Mother Postpartum Coparenting Relationship |  | 0.125 | -0.137 |
| Partner Postpartum Relationship Satisfaction |  |  | **-0.465** |

**2.9.** **Supplementary Table 9**

Correlation between the variables in the regression model predicting partner's Postpartum Coparenting Relationship

| Variables | Partner Antenatal Coparenting Relationship | Mother’s Postpartum Coparenting Relationship | Partners Postpartum Relationship Satisfaction | Partner Postpartum Anxiety at 12-weeks |
| --- | --- | --- | --- | --- |
| Partner Antenatal Coparenting Relationship | 1 | -0.176 | -0.082 | 0.277 |
| Mother’s Postpartum Coparenting Relationship |  | 1 | 0.125 | -0.137 |
| Partners Postpartum Relationship Satisfaction |  |  | 1 | **-0.465** |
| Partner Postpartum Anxiety Symptoms at 12-weeks |  |  |  | 1 |
